# Substance P-driven feed-forward inhibitory activity in the mammalian spinal cord

**DOI:** 10.1186/1744-8069-1-20

**Published:** 2005-06-29

**Authors:** Terumasa Nakatsuka, Meng Chen, Daisuke Takeda, Christopher King, Jennifer Ling, Hong Xing, Toyofumi Ataka, Charles Vierck, Robert Yezierski, Jianguo G Gu

**Affiliations:** 1 McKnight Brain Institute, University of Florida, Gainesville, Florida 32610, USA; 2 Department of Oral & Maxillofacial Surgery and Diagnostic Sciences, College of Dentistry, University of Florida, Gainesville, Florida 32610, USA; 3 Department of Orthodontics, College of Dentistry, University of Florida, Gainesville, Florida 32610, USA; 4 Department of Neuroscience, College of Medicine, University of Florida, Gainesville, Florida 32610, USA; 5 Comprehensive Center for Pain Research, University of Florida, Gainesville, Florida 32610, USA

## Abstract

In mammals, somatosensory input activates feedback and feed-forward inhibitory circuits within the spinal cord dorsal horn to modulate sensory processing and thereby affecting sensory perception by the brain. Conventionally, feedback and feed-forward inhibitory activity evoked by somatosensory input to the dorsal horn is believed to be driven by glutamate, the principle excitatory neurotransmitter in primary afferent fibers. Substance P (SP), the prototypic neuropeptide released from primary afferent fibers to the dorsal horn, is regarded as a pain substance in the mammalian somatosensory system due to its action on nociceptive projection neurons. Here we report that endogenous SP drives a novel form of feed-forward inhibitory activity in the dorsal horn. The SP-driven feed-forward inhibitory activity is long-lasting and has a temporal phase distinct from glutamate-driven feed-forward inhibitory activity. Compromising SP-driven feed-forward inhibitory activity results in behavioral sensitization. Our findings reveal a fundamental role of SP in recruiting inhibitory activity for sensory processing, which may have important therapeutic implications in treating pathological pain conditions using SP receptors as targets.

## 

Feedback/feed-forward inhibitory modulation driven by glutamate has been well studied in the dorsal horn of the spinal cord [1-3]. Little is know whether feedback/feed-forward inhibitory active may be driven in a glutamate-independent manner. A number of neuropeptides including substance P (SP) are also released from nociceptive primary afferent fibers [4]. SP has been regarded as a pain substance for decades [5-7], as supported by studies, including chemical ablation of lamina I neurons expressing the SP receptors [8], genetic disruption of the genes encoding substance P [9] and its receptors [10]. The nociceptive function of SP is mainly attributed to the activation of NK1 receptors (NK1R) that are expressed on nociceptive projection neurons located in lamina I of the dorsal horn [8,11,12]. It is unknown whether endogenously released SP can directly drive, in a glutamate-independent manner, inhibitory activity within the spinal cord to control nociceptive responses.

We performed patch-clamp recordings from dorsal horn neurons in lamina V (Figure 1a), a region important for nociceptive transmission and modulation [1,2]. When primary afferent fibers (dorsal roots) were briefly stimulated electrically (500 μA, 5 stimuli in 2.5 sec), EPSCs (excitatory postsynaptic currents) were recorded from lamina V neurons (Figure 1b). All EPSCs were blocked by ionotropic glutamate receptor antagonists 20 μM CNQX plus 50 μM APV (Figure 1b) or 3 mM kynurenic acid [3]. Brief stimulation of primary afferent fibers also evoked IPSCs (inhibitory postsynaptic currents). These immediate IPSCs (Figure 1c top) were driven by glutamatergic synaptic input, or glutamate-driven feed-forward inhibitory activity, because they were completely abolished in the presence of CNQX plus APV (Figure 1c bottom). However, when prolonged stimulation was applied (500 μA, 20 Hz, 1 min), a robust and long-lasting increase of IPSC frequency and amplitude was recorded in the presence of CNQX plus APV (n = 5, Figure 1d–f) or kyurenic acid (KA, n = 7, Figure 2c). These results revealed a feed-forward inhibitory (FFI) pathway not driven by glutamate.

**Figure 1:**
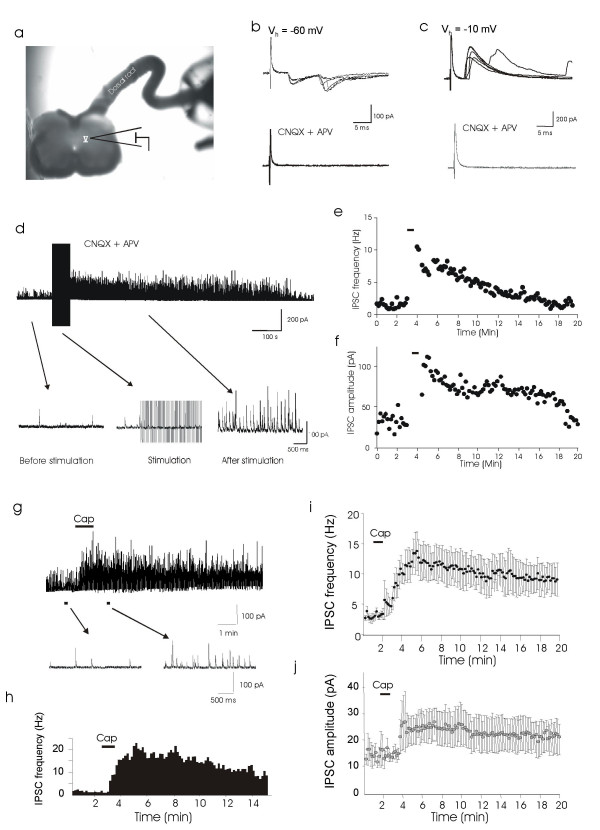
**Feed-forward inhibitory activity in the absence of glutamatergic driving force**. **a**, Rat spinal cord slice with attached dorsal root. A portion of the root is sucked into a stimulation electrode. Recordings were made in lamina V. **b**, Five consecutive traces show EPSCs evoked by electrical stimulation (top). The EPSCs were abolished in the presence of 20 μM CNQX plus 50 μM APV (bottom). V_h _= -60 mV. **c**, In the same cell, stimulation evoked IPSCs (top), which were abolished in the presence of 20 μM CNQX and 50 μM APV (bottom). V_h _= -10 mV. **d**, In the same cell, trains of stimulation (20 Hz for 1 min) increased IPSCs in the presence of 20 μM CNQX plus 50 μM APV. Top trace was IPSCs recorded before and after electrical stimulation. The bottom 3 traces are at an expanded scale. V_h _= -10 mV. **e&f**, Time course of IPSC frequency (**e**) and amplitude (**f**). Horizontal bars indicate stimulation. Overall, at peak responses, IPSC frequency increased to 376 ± 47% of control (n = 5, P < 0.05); IPSC amplitude increased to 228 ± 74% of control (n = 5, P < 0.05). Similar results were also obtained in the presence of 3 mM kynurenic acid (see Figure 2c). **g–j**, Capsaicin-induced increases in inhibitory activity in the absence of glutamatergic driving force. **g**, The top trace is a continuous recording of IPSCs from a rat lamina V neuron before and following the application of 2 μM capsaicin in the presence of 3 mM kynurenic acid. The bottom 2 traces are at an expanded scale. **h**, The time course of IPSC frequency in (**g**). bin width: 10s. **i&j**, Capsaicin-induced increases in IPSC frequency (**i**) and amplitude (**j**) recorded from 6 rat lamina V neurons in the presence of 3 mM kynurenic acid.

**Figure 2:**
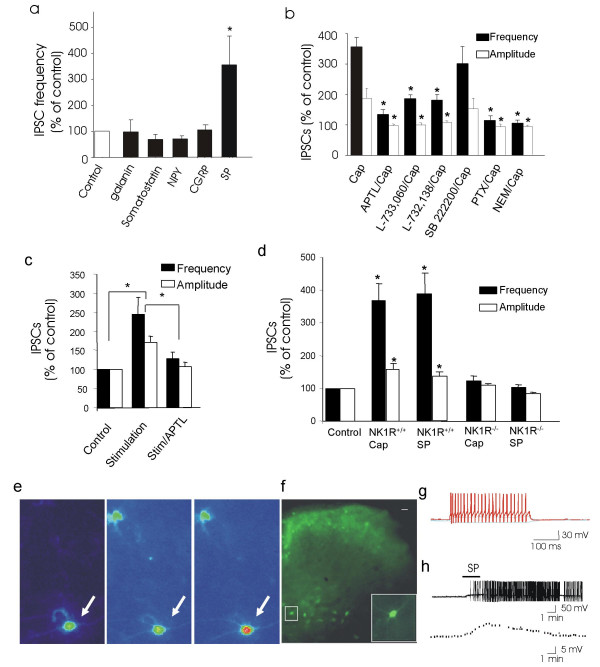
**Feed-forward inhibitory activity driven by SP through NK1 receptor activation**. **a**, Effects of exogenously applied neuropeptides on IPSCs recorded in rat lamina V neurons. Neuropeptides tested include galanin (0.3 μM, n = 4), somatostatin (2 μM, n = 4), NPY (1 μM, n = 4), CGRP (0.5 μM, n = 5), and substance P (1 μM, n = 6). **b**, Antagonism of capsaicin-induced increases in inhibitory activity in rat lamina V neurons. Capsaicin was applied in the presence of neurokinin receptor antagonists APTL (10 μM, n = 5), L-733, 060 (2 μM, n = 4), L-732,138 (100 μM, n = 5), SB222200 (2 μM, n = 8), and the Gi/o protein blockers pertussis toxin (2 μg/ml, n = 4) and NEM (100 μM, n = 5). **c**, Antagonism of electrical stimulation-induced increases in inhibitory activity by the SP antagonist APTL (10 μM, n = 7). Recordings were from rat lamina V neurons. **d**, Effects of capsaicin and SP on IPSCs in NK1R^+/+ ^mice (n = 8 for capsaicin, n = 10 for SP) and NK1R^-/- ^mice (n = 21 for cap, n = 11 for SP). Experiments were performed in the presence of 3 mM kynurenic acid (**a–d**) or 20 μM CNQX plus 50 μM APV (some experiments in **a**&**b**). **e**, Images show a cultured GIN mice EGFP neuron (arrow indicated) before (left) and after loading the Ca^2+ ^indicator Fluor-3 (middle), and following application of 100 nM SP (Right). The experiment was performed in the presence of 500 nM TTX and 3 mM kynurenic acid. Similar results were obtained from 22 EGFP neurons. **f**, The florescence image shows a spinal cord slice obtained from a GIN mouse. An EGFP neuron in lamina V is indicated by a small box and enlarged in a bigger box. **g**, Non-adaptive action potential firing induced by depolarizing current (50 pA) in the EGFP neuron. **h**, Application of 1 μM SP produced a prolonged depolarization and action potential firings (top) in the same cell. The dotted line (bottom) shows, at expanded scale, the membrane depolarization (action potentials are omitted for clarity). Kynurenic acid (3 mM) was present throughout the experiments (n = 7).

We used capsaicin, the active ingredient of hot chili peppers, to stimulate primary afferent fibers. Capsaicin is widely used as a natural stimulant for studying nociception. It excites nociceptive primary afferent fibers to release glutamate and neuropeptides including substance P through activation of TRPV1 receptors [13-15]. Capsaicin (2 μM) produced a robust and long-lasting increase in IPSC frequency and amplitude in the presence of 3 mM kynurenic acid (Figure 1g–j). The capsaicin effects were similar in the presence of kynurenic acid or other glutamate receptor antagonists (Additional file: 1, Figure 1a–c), indicating that the effects were unlikely due to an incomplete block of glutamate-driven FFI. Inhibitory neurons in lamina V use both GABA and glycine as co-transmitters [16], and increases of IPSCs by capsaicin were completely abolished in the presence of 20 μM bicuculline and 2 μM strychnine (n = 8).

It is unknown whether, transmitters, other than glutamate released from primary afferent fibers can directly drive inhibitory circuitry in the spinal cord. If a transmitter can drive FFI, exogenous application should increase inhibitory activity. We examined neuropeptides thought to be released from primary afferent fibers. Galanin (300 nM), NPY (neuropeptide Y, 1 μM), somatostatin (2 μM), and CGRP (calcitonin gene-related peptide, 0.5 μM) were tested, but none increased IPSCs (Figure 2a). However, SP significantly increased inhibitory activity under conditions when ionotropic glutamate receptors were blocked; SP increased IPSC frequency to ~350% of control (Figure 2a, n = 6) and amplitude to ~200% of control (n = 6).

If endogenously SP drives FFI following capsaicin stimulation, SP receptor antagonists should attenuate FFI. APTL (D-Arg1, D-Pro2, D-Trp7,9, Leu11]-Substance P, 10 μM), a neurokinin receptor antagonist, substantially blocked capsaicin-induced increases in IPSCs (Figure 2b). L-733,060 (2 μM) and L-732,138 (100 μM), two NK1 receptor (NK1R) antagonists, also inhibited capsaicin-induced increases in IPSCs. NK3 receptors are expressed in the dorsal horn, but SB22200 (2 μM), a NK3 receptor antagonist, did not significantly attenuate capsaicin-induced increases of IPSCs. Similar to capsaicin stimulation, we found that FFI elicited by electrical stimulation was largely abolished by the NK1R antagonist ATPL (Figure 2c). These results suggest that endogenous SP drives inhibitory activity.

NK1Rs couple with either the pertussis toxin (PTX)-insensitive Gq/G_11 _family [17] or PTX-sensitive Gi/Go family depending on cell types [18,19]. To elucidate which type of G-proteins was involved in SP-driven FFI, PTX was tested. We found that capsaicin-induced increases in inhibitory synaptic activity were completely abolished when spinal cord slices were pretreated with PTX (Figure 2b). Capsaicin-induced increases of inhibitory synaptic activity were also completely blocked in the presence of NEM (N-Ethylmaleimide), a Gi/Go protein inhibitor (Figure 2b). Thus, PTX-sensitive G-protein is involved in SP-driven FFI.

To confirm the involvement of NK1Rs, we used spinal cord slice preparations obtained from both wild type (NK1R^+/+^) and NK1R knockout mice (NK1R^-/-^). While capsaicin increased IPSCs in NK1R^+/+ ^mice, it had no effect in NK1R^-/- ^mice (Figure 2d). Consistent with this result, SP (1 μM) did not increase IPSCs in NK1R^-/- ^mice, but did substantially increase IPSCs in NK1R^+/+ ^mice (Figure 2d, Additional file: 1, Figure 2a,b). Thus, endogenous SP released from primary afferent fibers drives inhibitory activity (SP-driven FFI).

Possible cellular mechanisms of SP-driven FFI include i) direct excitation of inhibitory neurons; ii) via intermediate steps; and/or iii) through synaptic modulation. If NK1Rs are expressed on dorsal horn inhibitory interneurons [20], SP may directly excite inhibitory neurons. To test this possibility, we used dorsal horn neuron cultures made from GIN mice, a strain of transgenic mice that express EGFP (enhanced green fluorescent protein) under control of a promoter for GAD67 [21]. In GIN mice, almost all EGFP neurons examined in the dorsal horn are inhibitory neurons [22]. As shown in Figure 2e, SP (100 nM) increased intracellular Ca^2+ ^in ~30% (23/77) of EGFP neurons tested in the presence of 500 TTX and 3 mM kynurenic acid. We determined whether EGFP neurons in lamina V responded to SP using spinal cord slices prepared from GIN mice (Figure 2f). Most EGFP neurons recorded (64%) showed non-adaptive action potential firing in response to membrane depolarization (Figure 2g). Of 22 EGFP neurons examined, 7 (~30%) responded to 1 μM SP with prolonged membrane depolarization (5 ± 1 mV, n = 7) and action potential firing (Figure 2h). These results suggest that a cellular mechanism of SP-driven FFI is direct excitation of inhibitory interneurons by SP.

We found that SP (Additional file: 1, Figure 3a–c) and capsaicin (n = 12) had no effect on mIPSCs. SP also did not affect paired-pulse eIPSC ratio and corresponding eIPSC ratio (Additional file: 1, Figure 3d–f). These results suggest that SP/NK1R-mediated increases of IPSCs represent feed-forward neuronal activity rather than pre- or post-synaptic modulation at inhibitory synaptic junction sites.

We evaluated the extent SP-driving inhibitory activity contributes to the total inhibitory activity under normal conditions, i.e. without blocking glutamate-driven FFI. We also compared temporal phases between SP-driven FFI and glutamate-driven FFI. In NK1R^+/+ ^mice, IPSC frequency and amplitude were increased after trains of electrical stimulation (Figure 3a,c,d), similar to the results when glutamatergic driving force was blocked (Figure 1a–f & Figure 2c). In contrast, in NK1R^-/- ^mice, IPSCs were not significantly changed after the same trains of stimulation (Figure 3b,c,d). We examined IPSCs during electrical stimulation and found that, in both NK1R^+/+ ^and NK1R^-/- ^mice, IPSCs were elicited pulse-by-pulse immediately following each stimulus. These immediate IPSCs represented glutamate-driven FFI because they could be blocked by ionotropic glutamate receptor antagonists (see Figure 1c). Since the pulse-by-pulse inhibitory activity was seen in both NK1R^+/+ ^and NK1R^-/- ^mice, but the long-lasting increases in IPSCs after trains of stimulation were only observed in NK1R^+/+ ^mice, it suggests that the latter is driven by substance P through NK1R activation. Similar to electrical stimulation, a large and long-lasting increase in inhibitory synaptic activity was observed in NK1R^+/+ ^mice but not in NK1R^-/- ^mice after capsaicin stimulation in the absence of ionotropic glutamate receptor antagonists (Figure 3e). Thus, SP-driven FFI and glutamate-driven FFI have distinct temporal phases.

**Figure 3:**
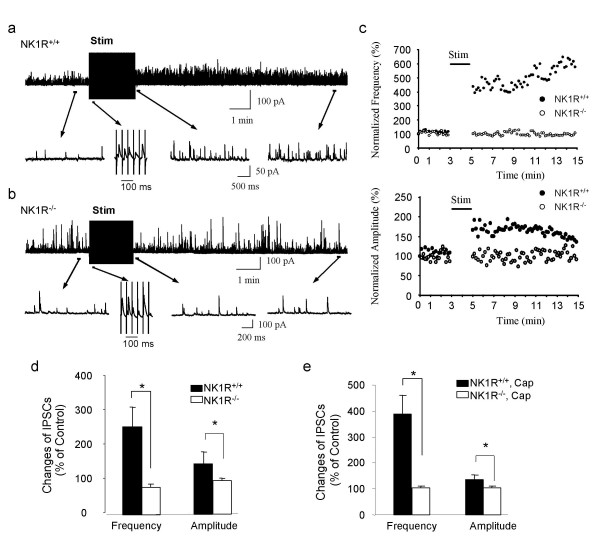
**SP-driven inhibitory activity under conditions when glutamatergic driving force is intact**. All experiments were performed in bath solution without glutamate receptor antagonists. **a**, A continuous recording of IPSCs from a lamina V neuron of a NK1R^+/+ ^mouse. Four traces (bottom) show, at an expanded scale, the IPSCs before, during, 1 sec after, and 9 min after trains of stimulation. The trace during stimulation is at a more expanded scale to show pulse-by-pulse eIPSCs. **b**, Same as **a **except the experiment was performed on a NK1R^-/- ^mouse. The pulse-by-pulse eIPSCs (second trace of lower panel) were similar to those of NK1R^+/+ ^mice, but ISPCs returned to the basal level immediately after termination of the train stimulation (third trace of lower panel). **c**, Time course of IPSC frequency (top) and amplitude (bottom). IPSCs during stimulation are not included. **d**, Peak IPSC frequency and amplitude after trains of stimulation in NK1R^+/+ ^(n = 6) and NK1R^-/- ^mice (n = 8). In **a–d**, stimulation was applied at intensity of 500 μA and a frequency of 20 Hz. **e**, Capsaicin-induced changes of IPSCs in lamina V neurons of NK1R^+/+ ^mice (n = 6) and NK1R^-/- ^mice (n = 16).

One physiological role of SP-driven FFI may be to balance neuronal activity and counteract SP-mediated nociceptive responses in the dorsal horn. To examine this potential physiological function, a behavioral model was used to see if blockade of SP-driven FFI, using an NK1R antagonist, causes behavioral sensitization to nociceptive stimuli. However, an NK1R antagonist will also block SP-mediated nociceptive response, thus interfering with the observation of a functional change following blockade of SP-driven FFI. To solve this complication, we chemically ablated NK1R-expressing neurons in the superficial lamina (Figure 4a&b); most of these neurons are nociceptive projection neurons responsible for SP-mediated nociception [8] and SP-evoked descending modulation [23]. Ablating NK1R-expressing neurons in the superficial lamina was achieved by intrathecally applying substance P-conjugated saporin (SP-SAP) [8], a targeted toxin for NK1R-expressing neurons (Figure 4a&b). In these animals, NK1R-expressing neurons in deep laminae remain intact or less affected [8,12,24]. To verify that SP-driven FFI remains intact, we used spinal cord slices prepared from SP-SAP treated animals and made recordings from lamina V neurons. Capsaicin was found to increase IPSCs to a similar degree in animals with (Figure 4c) or without SP-SAP treatment (Figure 1g–j, Supplementary Figure 1), indicating that the ablation did not affect SP-driven FFI in lamina V.

**Figure 4:**
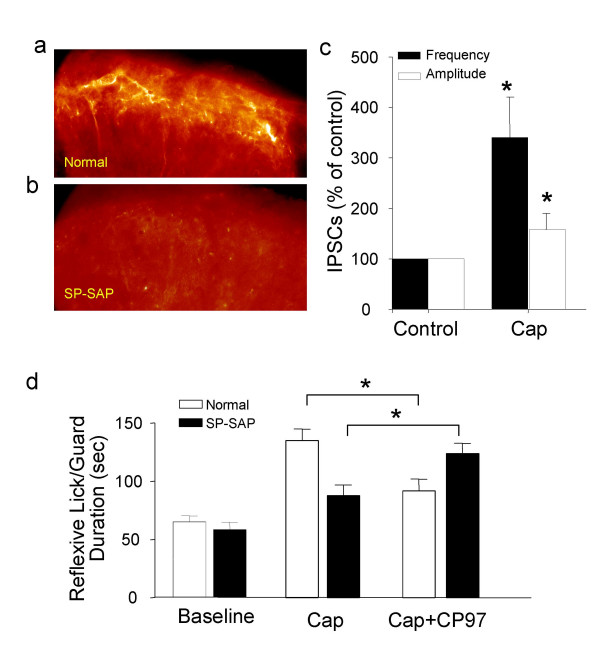
**Assessment of the role of SP-driven inhibitory activity in behavioral responses to noxious stimuli**. **a&b**, Micrographs show NK1 receptor immunoreactivity in the lamina I region of a normal rat (**a**) and 14 days following intrathecal application of SP-SAP (**b**). **c**, Capsaicin-induced increases of IPSCs recorded from lamina V neurons of SP-SAP treated rats (n = 5). **d**, The first set of bars show baseline of reflexive lick/guard response to heat stimuli at 44.5°C in normal (open bar, n = 8) and SP-SAP rats (solid bar, n = 8). The second set of bars show sensitization of behavioral responses by capsaicin in normal rats (n = 8) and attenuation of the behavioral response in SP-SAP rats (n = 8). The third set of bars show that the NK1 receptor antagonist CP97 attenuated behavioral responses in normal rats (n = 8) but sensitize behavioral response in SP-SAP rats (n = 8) (see Additional file: 1).

SP-SAP treated animals were used to access if the SP-driven FFI plays a role in controlling nociceptive behavioral responses. Reflexive lick/guard (L/G) responses to nociceptive heat stimuli at 44.5°C [24] were determined. Both the control and SP-SAP groups showed similar baseline responses to noxious stimuli (Figure 4d) [8]. Control rats showed behavioral sensitization following application of capsaicin cream to the planter surface, but a substantial attenuation of behavioral sensitization was observed in parallel experiments carried out in SP-SAP animals [8]. To examine whether the NK1-expressing neurons in deeper laminae of SP-SAP animals may intrinsically control behavioral responses to nociceptive heat stimuli, the behavioral responses were determined following blockade of NK1Rs by its antagonist CP-96,345 (36 nmol). Nociceptive reflexes showed sensitization when CP-96,345 was applied in SP-SAP animals, but behavioral hypersensitivity was attenuated by CP-96,345 in control animals (Figure 4d). The opposite effects of NK1R antagonists between normal and SP-SAP animals indicate a dual function of NK1Rs in nociceptive processing *in vivo*. The behavioral sensitization by the NK1R antagonist in SP-SAP animals revealed a role of SP-driven FFI in controlling nociceptive responses.

SP-driven FFI is a novel sensory processing mechanism. The unique feature is its temporal phase that extends long time after stimulation. This is distinct from glutamate-driven feedback/feed-forward inhibitory activity. Compromising SP-driven FFI can result in sensory hypersensitivity, providing implications in sensory pathology and therapeutics that targets neurokinin system [8,12].

## Methods

Electrophysiology recordings were performed on lamina V neurons in transverse spinal cord slices prepared from rats, NK1R^+/+ ^and NK1R^-/- ^mice, and GIN mice. Sprague Dawley rats were used at the age of 35 ± 7 days. Balb/c NK1R knockout mice (NK1R^-/-^) and GIN mice [21] (Jackson Laboratory) were used at the age of 21–35 days. Transverse slices were sectioned (600 μm in thickness) from spinal cord L5 segments of these animals [25]. In each experiment, a spinal cord slice was transferred to a recording chamber. The slice was superfused with a bath solution containing (in mM) 117NaCl, 3.6KCl, 2.5CaCl_2_, 1.2MgCl_2_, 1.2NaH_2_PO_4_, 25NaHCO_3_, and 11glucose, equilibrated with 95% O_2 _and 5% CO_2_, pH 7.35, 24°C. For voltage-clamp recordings, electrodes (~5 MΩ) were filled with a solution containing (in mM): Cs_2_SO_4 _110, CaCl_2 _0.5, MgCl_2 _2, Tea-Cl 5, EGTA 5, HEPES 5, pH 7.2. For current-clamp recordings, electrodes were filled with a solution containing (in mM): potassium gluconate 120, KCl 20, MgCl_2 _2, Na_2_ATP 2, NaGTP 0.5, HEPES 20, EGTA 0.5, pH 7.2. In experiments to determine EPSCs, cells were held at -60 mV. When IPSCs were recorded, cells were held at -10 mV. Unless otherwise indicated, IPSCs were recorded in the presence of 3 mM kynurenic acid. Miniature IPSCs (mIPSCs) were recorded in the presence of 500 nM TTX.

To stimulate primary afferent fibers, capsaicin (2 μM) was bath applied for 1 min. Capsaicin-induced increases in inhibitory activity were characterized pharmacologically with APTL (D-Arg1, D-Pro2, D-Trp7,9, Leu11]-Substance P, 10 μM), L-733, 060 (2 μM), L-732,138 (100 μM), SB222200 (2 μM), pertussis toxin (PTX, 2 μg/ml), NEM (N-Ethylmaleimide; 100 mM). Except for PTX, all compounds were applied through bath solution; all antagonists and blockers were pre-applied for 10 min. In experiments using PTX, spinal cord splices were pretreated with 2 μg/ml PTX for 2–4 hours.

To elicit feed-forward inhibitory activity by electrical stimulation, dorsal roots were stimulated electrically through a suction electrode. Stimulation was applied at an intensity of ~500 μA and pulse duration of 100 μsec. Unless otherwise indicated, stimulation was applied in a train of pulses that had a frequency of 20 Hz and duration of 1 min. Recordings were performed in the bath solution containing (in mM) 117 NaCl, 3.6 KCl, 4 CaCl_2_, 0.5 MgCl_2_, 1.2 NaH_2_PO_4_, 25 NaHCO_3_, 11 glucose, equilibrated with 95% O_2 _and 5% CO_2_.

To examine whether SP had effects on evoked IPSCs, paired-pulse evoked IPSCs were examined before and following application of 1 μM SP. Paired-pulse evoked IPSCs were elicited by focal stimulation in lamina V near the recorded neurons. Stimuli were applied at the intensity of 50–150 μA, pulse duration of 100 μs, and paired-pulse interval of 100 ms. The interval between two sets of paired-pulses was 10 s.

Calcium Imaging was performed on dorsal horn neuron cultures (5–7 days) made from neonatal GIN mice [26]. Cells were perfused with bath solution containing (in mM): 150 NaCl, 5 KCl, 2 MgCl_2_, 2 CaCl_2_, 10 glucose, 10 HEPES, pH 7.4; 500 nM TTX and 3 mM kynurenic acid. EGFP neurons were first identified and an image was taken. Cells were then loaded with the Ca^2+ ^indicator Fluo-3 on the stage of microscope. Subsequently, calcium imaging was performed [27], the effect of SP (100 nM) on EGFP neurons was tested.

To chemically ablating NK1R-expressing lamina I neurons with SP-SAP [8,24], a 32 g catheter was inserted into the lumbosacral subarachnoid space (L6-S1) of adult rats (250–300 g) [28] and SP-SAP (300 ng, substance P-conjugated saporin, Advanced Targeting System) was injected through the catheter to the lumbar enlargement. Fourteen days after this procedure, animals were used for *in vitro *electrophysiological recordings or *in vivo *behavioral tests. Controls were animals after sham operation.

Behavioral tests were performed on 8 SP-SAP treated animals and 8 control animals. Reflexive lick/guard responses were assessed in two consecutive ten-minute trials involving 36.0°C (pre-test) trial and then a 44.5°C (test) [24,29]. Lick responses were defined as a stereotyped lifting of the hindlimb followed by holding and licking the hindpaw. Guard responses were defined as an exaggerated raising of the hindlimb. Peripheral sensitization of behavioral responses was induced by application of capsaicin cream (1%) to the planter surface of one hindpaw. Reflexive responses were assessed three hours after application. To test the effects on behavioral responses following blockade of NK1 receptors, CP-96,345 (36 nmol), an NK1 antagonist was applied through the catheter 10 min before behavioral tests.

NK1 receptor immunostaining was performed after behavioral tests to confirm the effective removal of NK1R-expressing lamina I neurons in SP-SAP treated animals. NK1R immunostaining was performed using a polyclonal anti-NK1R serum (1:3000) on a series of sections (100 μm in thickness) cut from L5 of the spinal cord.

Analysis of synaptic events, including threshold setting and peak identification criteria, were performed according to a method previously described [26]. For calcium imaging experiments, responsive neurons are defined as ΔF/Fo > 20%. The duration of behavioral responses were collected by custom software (EVENTLOG) across testing sessions for all rats [24,29]. Unless otherwise indicated, data represent Mean ± SEM, * p < 0.05, student-t test. Statistical analysis of behavioral responses was performed by ANOVA, followed by Newman-Keuls post-tests.

## Supplementary Material

Additional file 1 Substance P-driven inhibitory activity in the mammalian spinal cord Click here for file
