# Supplementary material for: Substance P-driven feed-forward inhibitory activity in the mammalian spinal cord
Source: Mol Pain. 2005 Jun 29;1:20. doi: 10.1186/1744-8069-1-20 (PMC1185563; doi:10.1186/1744-8069-1-20)
Supplement: Additional file 1 — Substance P-driven inhibitory activity in the mammalian spinal cord [file 1744-8069-1-20-S1.doc]

**Substance P-driven inhibitory activity in the mammalian spinal cord: Supplementary Figures**

Terumasa Nakatsuka, Meng Chen, Daisuke Takeda, Christopher King, Jennifer Ling, Hong Xing, Toyofumi Ataka, Norma Gerard, Charles Vierck, Robert Yezierski & Jianguo G. Gu

**Supplementary Figure 1. Capsaicin-induced increases of inhibitory activity in the presence of different ionotropic glutamate receptor antagonists**

**a&b,** Pooled results show the peak IPSC frequency (**a**) and peak IPSC amplitude (**b**) following application of 2 mM capsaicin. Capsaicin was applied in the presence of 3 mM kynurenic acid (n = 6), 20 mM CNQX plus 50 mM APV (n = 16), or 20 mM CNQX plus 50 mM APV and 100 mM GYKI 52466 (GYKI, n = 7). **c,** Results from E&F are expressed as percent of controls. Controls were IPSC frequency or amplitude before capsaicin and were scaled as 100% (first set of bars). Data represent Mean ± SEM, * p < 0.05, paired Student-t test.

**Supplementary Figure 2. Effects of SP on IPCSs of lamina v neurons in NK1R+/+ and NK1R-/- mice**

Experiments were performed in the presence of 3 mM kyneurenic acid. **a,** Time course of IPSC frequency in NK1R+/+ (n = 6) and NK1R-/- mice (n = 5). **b,** Time course of IPSC amplitude in NK1R+/+ (n = 6) and NK1R-/- mice (n = 5). Amplitude scale on the left is for NK1R+/+ and the scale on the right is for NK1R-/- mice. All recordings were made from lamina V neurons. SP was applied at concentration of 1 μM for 1 or 3 min (arrow indicated).

**Supplementary Figure 3. Lack of effect by SP on mIPSCs and eIPSCs**

**a,** Two sample traces show mIPSCs recorded from rat lamina V neurons before (control) and following 1 mM SP. **b**, Cumulative probability of inter-event intervals (left) and amplitude (right) from the same neurons in (**a**). **c**, Pooled results show no effect of SP on mIPSCs (n = 5). **d,** Sample traces recorded from a rat lamina V neuron show IPSCs evoked by paired-pulse stimulation before (control) and following 1 mM SP. **e&f** Pooled results (n = 19) show no change of the paired-pulse IPSC ratio (**e**) and corresponding IPSC ratio (**f**).
